# The worldwide investigating nurses’ attitudes towards do-not-resuscitate order: a review

**DOI:** 10.1186/s13010-021-00103-z

**Published:** 2021-09-07

**Authors:** Neda Raoofi, Samira Raoofi, Rostam Jalali, Alireza Abdi, Nader Salari

**Affiliations:** 1 Department Nursing, Nursing and Midwifery Faculty, Kermanshah University of Medical Sciences, Kermanshah, Iran; 2 Health Care Management, Iran University of Medical Sciences, Tehran, Iran; 3 Faculty of Nursing and Midwifery, Kermanshah University of Medical Sciences, Kermanshah, Iran

**Keywords:** Do-Not-Resuscitate Order, DNR Order, Nurses, Systematic Review, Iran, Attitudes

## Abstract

**Background:**

The acceptance or practical application of the do-not-resuscitate order is substantially dependent on internal or personal factors; in a way that decision-making about this issue can be specific to each person. Moreover, most nurses feel morally and emotionally stressed and confused during the process decision-making regarding DNR order. Therefore, the purpose of the present study was to evaluate nurses’ attitudes towards DNR order in a systematic review.

**Methods:**

This critical survey was conducted using a systematic review protocol. To this end, the most relevant articles published in domestic and foreign databases with no time limits until October 2018 were searched. The inclusion and exclusion criteria were articles on DNR order, studies about nurses’ attitudes, descriptive and analytical research papers, as well as those with download links and full texts. The given articles were also assessed in terms of their quality and their main results were extracted.

**Results:**

Of the total number of 1663 articles searched in the process of systematic review to investigate nurses’ attitudes towards DNR order, 88 articles were included in the full-text review step and finally 10 articles, meeting the inclusion criteria, were found. Assessing the quality of articles included in this review showed that 8 articles, in general, were of good quality and 2 studies were characterized with moderate quality. The main factors associated with nurses’ attitudes towards DNR order were grouped into three categories of (1) nurses’ attitudes towards DNR order, (2) guidelines for DNR order, and (3) decision-making by patients and their families about DNR order. In most of the studies examined, nurses’ attitudes towards DNR order were reported positive.

**Conclusion:**

It seemed that nurses were willing to get involved in DNR order and each hospital was recommended to develop a written DNR policy directing individuals and avoiding their confusion in this respect.

## Background

Today, with the increase in the quality of medical care and the improvement of the welfare of society, the number of patients in the final stages of chronic diseases is increasing day by day. Therefore, providing care for such patients can bring about numerous challenges including doing or not doing cardiopulmonary resuscitation (CPR). It should be noted that CPR contains all primary and advanced therapeutic actions in the conditions of cardiac arrest due to various clinical reasons [1, 2]. Moreover; CPR can induce favorable results in some cases, but it can sometimes end in failure. Even though survival rate is defined as coming back to long and high-quality life with no annoying problems and disabilities, so, the percentage of successful CPRs will be rare [3]. Accordingly, about half a century ago, DNR order was introduced in medical texts and the first guidelines for this procedure were released following the ineffectiveness of CPR in most cases along with imposed heavy costs in terms of financial expenses and waste of human services [4–7].

Sometimes, medical teams do not consider CPR as a useful activity due to patients’ general conditions, their age, functional status in cardiac arrest, distance between cardiac arrest and onset of CPR, as well as underlying illnesses and their prognosis [7] In majority of cases, patients prefer to opt for DNR order with regard to existing conditions and complications [8].

Given the emergency of providing care services in this situation as well as lack of patient clinical capacity to make informed decisions, occurrence of emotionally anxious reactions by patients’ companions and absence of specified clinical guidelines in such cases, physicians might feel confused in the process of decision-making about doing or not doing CPR. This confusion sometimes results in inappropriate decisions and patients who could benefit from CPR may be deprived of this care service and those willing to receive it might have a short life in intensive care unit (ICU) accompanied by major physical problems and mental stresses [3].

Dealing with this issue is not the same in various societies considering the diversity of religious beliefs, rituals and customs, cultures, and as well as socioeconomic status [3]. In this domain, culture is taken into account as one of the very important factors [9]. In spite of numerous studies on DNR order across the world, physicians and medical teams are still facing challenges in this regard. DNR order is not considered as a barrier to performing medical interventions and nursing care services [9–11]. It should be noted that patients with DNR order receive all care services such as venous therapy, antibiotics, painkillers, and pain relievers [9, 12, 13].

Despite unprecedented advances in technologies associated with care services, nurses are still engaged in taking care of patients with DNR order, so there is the possibility of involvement or non-involvement of nurses in the process of DNR order [14]. Considering the close relationship between nurses and patients and their families, no involvement in DNR order by nurses can frequently initiate feelings of anger, anxiety, and frustration in this group [15–18].

Once decisions are made in this domain, nurses left alone with the consequences caused by decision-making about patient and family care. Lack of clear descriptions for patient care can be mostly influenced by nurses’ DNR order and also question the suitability of this decision as well as the benefit of providing specific services for patients with this order [19–22]. These conditions can predominantly confuse nurses that had already encountered with patients expected to die but survived following CPR and returned back to their normal life [23–25].

Problems that occur in the face of DNR order can fall into intrapersonal and interpersonal ones. In this regard, intrapersonal difference is caused by a conflict between individual values in encounters with DNR order and quick interruption of care services considering long-term sufferings in patients. Interpersonal controversies also take place when nurses’ attitudes are different from those involved in the process of DNR [23, 25, 26]. In ICUs, wherein the main objective is maintaining vital physiological functioning, DNR order is also considered as a complicated and multi-faceted event that can challenge nurses. So, nursing staff working in ICUs acknowledge that DNR order can mostly instigate ethical problems in providing care services. Accordingly, nurses are likely to concentrate on patients’ families and consequently show more flexibility in terms of visits with patients as well as presence of families at the bedside [27].

Considering religious values and beliefs, this issue needs to be delineated all over the world in order to reduce confusion in medical teams at the bedside. Therefore, the purpose of the present study was to investigate nurses’ attitudes towards DNR order in Iran and across the world in a systematic review.

## Methods

**Fig. 1:**
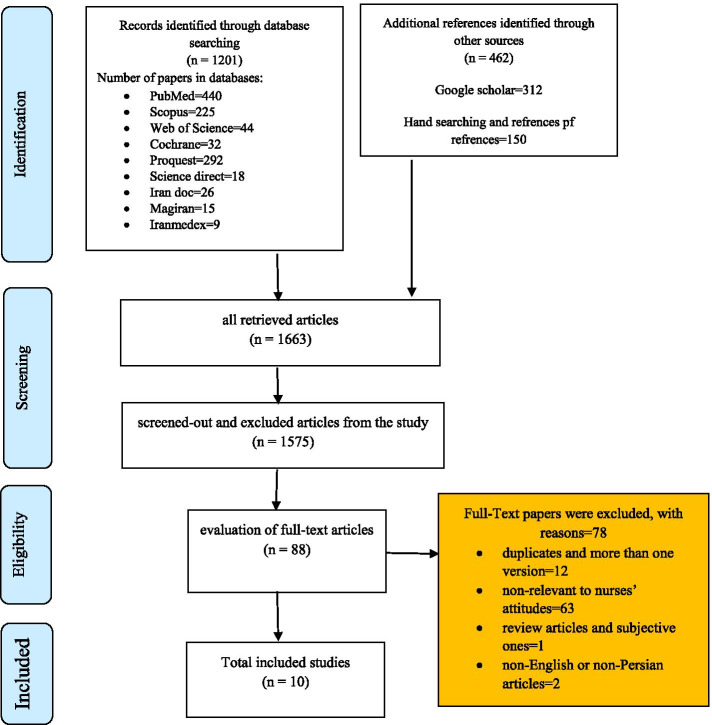
Flow diagram of our review process (PRISMA)

This critical survey was conducted using a systematic review protocol. The statistical population included all the articles on DNR order among nurses in Iran and around the world. The search strategy was also fulfilled based on proper combination of Persian and English keywords (i.e. cardiopulmonary resuscitation, CPR, nurses, attitudes, do-not-resuscitate, do-not-resuscitate policy, do-not-resuscitate decision, do-not-resuscitate order, do not attempt resuscitation, do not attempt resuscitation order, DNR) and the studies and scientific documents were searched according to the features of search engines or databases. To conduct the given search; the databases of ScienceDirect, Web of Science, Cochrane, ProQuest, Scopus, PubMed, SID, Irandoc, Magiran, and Iranmedex were explored with no time limits to find the related articles. Moreover, the search was carried out in Google Scholar and the website of American Nurses Association (ANA). Furthermore, the references of the selected studies were reviewed to find articles missed in the search process. The search continued on articles published without any time limits until September 2018. Using the notification systems of online databases and Google Scholar, the search for articles was updated until October 2018. The inclusion and exclusion criteria in this study were articles related to DNR order, nurses’ attitudes, descriptive and analytical studies, and those with download links and full texts. Following the search and based on the specified keywords, the duplicates were initially removed and the remaining articles were selected through screen-outs based on titles, abstracts and full texts, considering the inclusion and exclusion criteria. Then, the full texts of the articles were retrieved from the databases upon their open access; and if not so, such articles were crossed out from the study. To assess the quality of the articles, they were reviewed by two individuals and comments by a third person were further used in case of disagreements. To validate and assess the quality of the given articles (validity of the methodology and the results of articles), critical tools matched with the type of study were employed. To review quantitative and qualitative studies, the Strengthening the Reporting of Observational Studies in Epidemiology (STROBE) and the Standards for Reporting Qualitative Research (SRQR) were also used, respectively. The selected articles were divided into three categories of good, medium, and weak ones in terms of quality. After removing duplicated and non-relevant articles, according to the article extraction form, the remaining ones were analyzed using thematic analysis. The findings were also reported in the form of descriptive tables containing author’s name, year and country of origin, summarized findings, and conclusions of each of the articles. The process of selecting articles and the number of searched, excluded, and included ones were illustrated in Fig. 1.

## Results

Of the 1663 articles retrieved from the online databases, a total number of 88 articles were examined. Considering the inclusion and exclusion criteria, 10 relevant articles were finally included in the systematic review and the remaining articles were excluded. Of the reviewed articles, 8 cases (80%) were of good quality and 2 articles (20%) were characterized with moderate quality. With regard to study location i.e. the country of origin in which the articles had been conducted, there was one relevant article in each country including Iran, Sweden, Poland, Belgium, Jordan, Australia, Japan, Canada, South Korea, and Rwanda examining nurses’ attitudes towards DNR order.

As presented in Table 1, 8 quantitative studies related to nurses’ attitudes and experiences regarding DNR order had been published in Iran, Canada, Poland, Belgium, Jordan, Australia, Japan, and South Korea between 1997 and 2014, respectively. Most studies (80%) were descriptive and cross-sectional ones.

**Table 1:** Characteristics of the articles included in the systematic review (*n* = 10)

Author’s name	Type of study	Year	Country of origin	Purpose of the study	Data collection instrument	Study setting	Main results
Goniewicz et al. [28]	quantitative	2011	Poland	emergency nurses’ attitudes towards implementation of DNR order	questionnaire	hospital emergency ward	67% of the respondents considered it necessary and obligatory to receive declarations or guidelines for DNR orders Nurses had adopted positive attitudes towards DNR order 7.3% of the participants stated that implementation of DNR msut be avoided 1. Most nurses believed that patients had the right to decide about DNR order 2. Existence of guidelines for DNR order is necessary 3. DNR order must be available in a written form 4. This procedure is considered as a social and moral problem
Al Khalaileh [29]	quantitative	2014	Jordan	Nurse’s attitudes and experiences concerning DNR order in Jordan	questionnaire	state-run hospitals (n = 3)	67% of the nurses recommended that patients’ families must be involved in making decisions about DNR order 81% of the nurses suggested a coded documentation system for nurses and physicians regarding decisions about DNR order 58% of the nurses agreed that there were standards and forms for DNR order 21% of the nurses agreed upon their involvement in decision-making about DNR order Finally, it was concluded that the nurses were willing to get engaged in DNR order and each hospital needed to develop a written DNR order directing individuals and preventing their confusion in this regard
De Gendt et al. [30]	quantitative	2000	Belgium	Investigating nurses’ attitudes towards decision-making about DNR orders in geriatric departments departments	questionnaire	hospital	74% of the nurses were involved in the process of DNR order 54% of the participants stated that DNA had not been implemented Nurses did not have a defined role in decision-making about DNR order. So, existence of standardized guidelines for decision-making about DNR seemed necessary
Manias. [31]	quantitative	1998	Australia	Experiences and attitudes of Australian nurses towards decisions concerning DNR order	questionnaire	hospital and emergency ward (n = 6)	Nurses had positive attitudes towards implementing DNR order Nurses believed that patients’ families, patients, and nurses needed to be involved in decision-making regarding DNR order Physicians were only responsible for making decisions about DNR order Existence of standardized guidelines for decision-making about DNR seemed necessary
Konishi [32]	quantitative	1997	Japan	Nurses’ attitudes towards DNR order policy in Japan	questionnaire	hospital and emergency ward (n = 6)	Almost all the nurses considered DNR order policy appropriate and supported it as an ideal process 85% of the nurses considered patients’ choices as decisive factors in implementation of DNR order 22% of the nurses put emphasis on DNR order 60% of the nurses stated that DNR order needed to be implemented as soon as possible Such a process could make nurses deal with difficult situations due to cultural and psychological factors
Moghadesian et al. [33]	quantitative	2014	Iran	attitudes among Nursing students at Tabriz and Kurdistan Universities of Medical Sciences towards DNR order	questionnaire	Schools of Nursing in the cities of Tabriz and Kurdistan	Nursing students participating in the study had negative attitudes towards DNR order Students believed that they needed to learn much more about this procedure It seemed that teaching students about DNR order could change their attitudes in this domain
Thibault-Prevost et al. [14]	quantitative	1997	Canada	Understanding ICU nurses’ attitudes towards DNR order	questionnaire	Nurses referring to College and Association of Registered Nurses of Alberta (CARNA)	Lack of instructions and legal guidelines for DNR orders Involvement of patients and families in making decisions about DNR order Nurse-physician communication and cooperation in the implementation of DNR order Existence of multilateral cooperation for making decisions to implement DNR order
Rye Park et al. [34]	quantitative	2008	South Korea	ICU nurses’ perceptions and attitudes towards DNR orders	questionnaire	hospital and ICU	96% of the participants considered the implementation of DNR order sometimes necessary 99.2% of the nurses considered explanations and descriptions of DNR order as a necessary issue 76.6% of the nurses agreed upon the implementation of DNR order 71% of the nurses considered patients’ preferences and wishes as the basis for implementation of DNR order 76.6% of the nurses agreed that decisions made about DNR orders needed to be based on guidelines for DNR order Only 22.2% of the nurses considered physicians’ votes as a basis for implementation of DNR order
Pettersson et al. [35]	qualitative	2014	Sweden	Nurses’ attitudes towards DNR order in the Department of Hematology and Oncology	semi-structured interview	hospital and department of hematology and oncology	In order to provide good nursing care services in relation to DNR order, it was necessary to pay attention to the following issues: 1. Clarification and documentation of DNR order 2. Giving awareness to patients’ relatives and family members about DNR order and involvement of their decisions and opinions
Nankundwa et al. [36]	qualitative	2017	Rwanda	Investigating ICU nurses’ experiences in Rwanda towards patients with DNR order	structured interview	hospital	Emotional anxiety: DNA order could induce emotional states in nurses and bring them into a difficult situation Prevention of good care: Existence of DNR order prevents good patient care services and it also affects numerous nursing activities Lack of guidelines for decision-making: in cases of implemented DNR order, only physicians had made such decisions Nurses believed that they would be allowed to decide about DNR order

The results of investigating quantitative articles in this systematic review showed that nurses had different attitudes towards DNR order.

In the study in Jordan, Al Khalaileh examined nurses’ attitudes and experiences concerning DNR order. In this study, 111 nurses working in three state-run hospitals were investigated. The findings revealed that 21% of the nurses had stated that they had the experience of involvement in decision-making regarding this procedure. Ultimately, it was concluded that such nurses were willing to play a part in DNR order [29].

In Belgium, De Gendt et al. explored nurses’ attitudes towards making decisions about DNR order in geriatrics departments and found that 74% of the nurses had been involved in the process of DNR order. Therefore, these participants acknowledged that they had the choice of DNR order and had also adopted positive attitudes towards this procedure [30].

In one other study by Moghadesian et al. conducted at two universities in the cities of Tabriz and Kurdistan, Iran, a total number of 186 nursing students were investigated and it was reported that nursing students had negative attitudes towards DNR order. However, these students stated that they needed to learn much more about this procedure. It seemed that teaching students about DNR order could change their attitudes in this regard [33].

The study conducted by Manias in Australia also showed that nurses had positive attitudes towards DNR order. Moreover, they put emphasis on involvement of patients’ families, patients, and nurses in decision-making concerning DNR order, since physicians were mostly responsible for such decisions. Finally, they reiterated that it was essential to have standard guidelines for decision-making about DNA order [31].

The results of quantitative studies included in this systematic review indicated that lack of guidelines for DNR order was one of the biggest barriers to implementing this procedure. For example, Goniewics et al. found that 67% of the respondents had assumed the existence of declarations or guidelines for DNR order as a necessary and obligatory issue [28]. Other main results of quantitative studies included in this systematic review were separately shown in Table 1.

Moreover, two qualitative studies investigating nurses’ attitudes and experiences towards DNR order were identified. These investigations had been conducted in Sweden and Rwanda in hospital environments between 2014 and 2017.

The main themes of the qualitative studies in this systematic review were role of guidelines in DNR order implementation, lack of a document registration and development system for DNR order, involvement of patients and their families in implementation of DNR order, ethical issues, as well as barriers to provision of proper care services [36] (Table 1).

## Discussion

The purpose of this systematic review was to evaluate nurses’ attitudes towards DNR order in Iran and across the world in a systematic review. After half a century, DNR order has been welcomed in many Central and Western European and North American countries and most of healthcare centers in these countries have developed specific policies for this procedure [37]. Studies conducted in recent years among healthcare staff in the United States, Finland, Sweden, and Germany also demonstrated that these individuals had adopted relatively positive attitudes towards DNR order despite the existence of some difference [38].

Moreover, in the Islamic culture in which life and human life have certain values and life moments are respected with high values, development of guidelines that have transparently elaborated the procedure to make decisions about DNR order and to reduce the interference of personal, impractical, and non-professional factors is of utmost importance. According to the related literature and despite the fact that DNR orders are implemented in some Muslim countries, it is not still legalized to do them. There are currently controversies regarding the legality of this decision in Iran and there is no definitive outcome; however, evidence has suggested that Iran’s legal system has the potential to regulate DNR order and its related issues [39].

The results of the analysis of the selected articles on nurses’ attitudes towards DNR order implied that such attitudes and perspectives could be different in terms of race, religion, country of origin, and other factors. But, in the end, the findings revealed that nurses in most articles had positive attitudes towards DNR order. In the study by Moghadesian et al. (2014) conducted at Tabriz and Kurdistan Universities of Medical Sciences in Iran, nursing students had a negative attitude towards DNR order. Furthermore, such students had stated that they needed to learn much more about this procedure. It seemed that teaching students about DNR order could change their attitudes in this regard [33] As well, studies by Al Khalaileh (2014) in Jordan [29], De Gendt et al. in Belgium [30], Manias (1998) in Australia [31], and finally those by Prevost et al. (2008) in Candida [14] and Rye Park et al. (1997) in South Korea [34] confirmed the presence of positive attitudes among nurses towards DNR order; that is, nurses and medical teams considered the implementation of DNR orders as a necessary issue. Unlike the studies mentioned, Goniewicz et al. in Poland investigating attitudes in a group of nurses towards DNR order and cases of making decisions about them found that 7.3% of the nurses had stated that implementation of DNA order needed to be avoided [28]. Additionally, the study by Konishi et al. (1997) examining nurses’ attitudes towards DNR order policy in Japan showed that almost all nurses had considered the implementation of DNR order as an appropriate procedure and had also supported it as an ideal process; however, such a procedure could pose a difficult situation for nurses due to cultural and psychological factors [32]. According to the studies conducted in different countries, there were a variety of attitudes towards DNR order and the basis for such an order depended on the country of origin and its culture, religious issues, guidelines and laws, psychological and mental issues of clinical staff, patients’ decisions and preferences, and other cases.

As well, almost all the studies indicated that it was necessary to implement DNR orders. Instructions or permits as well as legal guidelines for DNR order were also of great importance. Moreover, the nurses participating in the analyzed studies stated that they needed to have the right to make decisions about DNR order given their involvement in such situations. The study conducted by Pettersson et al. (2014) in Sweden similarly showed that it was necessary to develop guidelines and to document DNR orders [35].

In another study by Nankundwa et al. in Rwanda examining a total number of 6 ICU nurses’ experiences concerning patients with DNR order through interviews, it was noted that only physicians had made decisions about DNR cases. Nurses participating in this study also believed that they needed to be allowed to decide about DNR orders. Additionally, existence of guidelines for such a procedure for the nursing group was necessary [36].

Furthermore, the study by Rye Park et al. (1997) on ICU nurses’ perceptions and attitudes towards DNR order in South Korea revealed that 76.6% of the nurses had agreed with the decisions to implement DNR orders based on guidelines and only 22.2% of them considered physicians’ votes as the basis for implementing the given procedure [34].

In other investigations, nurses also considered the existence of instructions or legal guidelines for DNR orders as an essential issue. Given the involvement of nurses in the process of DNR orders, presence of guidelines for DNR orders could prevent confusion and other factors such as psychosocial issues in nurses, legal issues, etc.

## Conclusion

It should be noted that deciding about DNR orders is a difficult process that can be affected by various factors. The results of the studies in this domain indicated that making decisions regarding the implementation of DNR order should not be based solely on the wishes of a particular person. In the mentioned studies, most of the nurses had stated that nurses, patients, and patients’ families were required to play roles in deciding about DNR orders, and thus their willingness and desires needed to be taken into account. For example, the findings of the study by Goniewicz et al. [1]. In Poland revealed that nurses had stated that they needed to play a role in DNR orders and also have the right to decide about it [28], since they had no defined role in this domain [30].

The results of the study by Manias (1998) on Australian nurses’ experiences and attitudes towards decisions about DNR order also revealed that nurses believed that patients’ families, patients, and nurses needed to get involved in making decisions about DNR orders, since physicians were only responsible for deciding about such orders in current circumstances [31]. In addition, in the studies by Prevost et al. [14] and Rye Park et al. [34]. And other investigations in this domain [14, 29, 32, 33, 36]; involvement of patients’ families, patients, and nurses in deciding about DNR orders was emphasized.

The results of this study showed that, nurses were willing to implement DNR orders in the last moments of patients’ life. It was also suggested to develop a DNR order policy in each hospital to avoid any confusion in this regard. Moreover, it was required to pay attention to nurses’ roles and their encounters at the bedside in such conditions and take necessary measures in policy-making in this domain.

## Data Availability

Datasets are available through the corresponding author upon reasonable request.

## Abbreviations

DNR: Do-not-resuscitate
CPR: Cardiopulmonary Resuscitations
ICU: Intensive Care Unit
STROBE: Strengthening the Reporting of Observational Studies in Epidemiology
SRQR: Standards for Reporting Qualitative Research
ANA: American Nurses Association
